# ORGANIZATIONAL PRACTICES FOR THE AGING WORKFORCE: DEVELOPMENT AND VALIDATION OF THE LATER-LIFE WORK INDEX

**DOI:** 10.1093/geroni/igz038.2807

**Published:** 2019-11-08

**Authors:** Max R Wilckens, Anne M Wöhrmann, Jürgen Deller, Mo Wang

**Affiliations:** 1 Leuphana University Lüneburg, Lüneburg, Germany; 2 University of Florida, Gainesville, Florida, United States

## Abstract

Most developed countries face an ageing workforce. Extended working lives require organizations to employ older individuals successfully. It is, however, widely untapped, which organizational practices drive successful employment of an older and age-diverse workforce, because adequate measure are missing. We hence develop the Later Life Work Index (LLWI) as a multi-faceted measure for researchers and practitioners, combining the detailed level of assessment relevant in practice with thorough conceptual coverage. We build on an empirically derived taxonomy of organizational practices developed by Wöhrmann et al. (2018). Proposed taxonomy is based on qualitative expert interviews and consists of nine dimensions covering age-inclusive organizational climate and leadership, as well as practices regarding work design, health management, individual development, knowledge management, transition to retirement, continued employment options, and health and retirement coverage. Within a first study, we developed an inventory to operationalize the intentionally broad LLWI construct. Items were iteratively developed and pretested with 30-42 German human resource managers. The final inventory consists of 100 Likert scaled, sufficiently reliable items. Within a second study among 600 managers and older workers in Germany, we confirmed the factor structure proposed by the LLWI, and ensured construct validity regarding similar scales (convergent validity) and individual level health and motivation outcomes (criterion validity). Discriminant validity is shown among the index dimensions, and regarding positive and negative affect. This paper enhances the understanding and quantitative assessment of organizational practices for later life work. We further plan to reduce the number of items to increase practicability of the measure.

